# A Case of Acute Aortoiliac Occlusive Disease Presenting as Cauda Equina Syndrome and Fournier´s Gangrene

**DOI:** 10.1155/2019/4027460

**Published:** 2019-04-17

**Authors:** Rosalba Paone, Pekka Romsi

**Affiliations:** Oulu University Hospital, Oulu, Finland

## Abstract

Aortoiliac occlusive disease presents itself more frequently as chronic claudication, erectile dysfunction, and absent femoral pulses. Its acute manifestation is less frequently encountered in a clinical practice; hence, it presents sometimes as a diagnostic challenge. We illustrate a case of acute aortoiliac occlusive disease presenting with spinal cord ischemia and gluteal and scrotal necroses, which was initially diagnosed and treated as spinal cord compression. In order to avoid misdiagnosis, careful examination of peripheral pulses of both lower limbs should always be part of the initial evaluation of cauda syndrome and paraplegia and when Fournier's gangrene is suspected.

## 1. Introduction

Thrombotic obliteration of the aortic bifurcation was first described by Leriche and Morel in 1924 [1]. Aortoiliac occlusive disease (AIOD) presents itself more frequently as chronic claudication, erectile dysfunction, and absent femoral pulses. Dyslipidemia, male gender, smoking, diabetes mellitus, and hypertension are the main risk factors. Acute manifestation of AIOD is less frequently encountered in the clinical practice; therefore, it may pose as a diagnostic challenge, even to expert physicians. An acute onset of symptoms is associated with considerable morbidity and mortality. Aortic saddle embolus or thrombosis of an atherosclerotic abdominal aorta is the most common cause of acute aortoiliac occlusion [2, 3]. The absence of femoral pulses on vascular examination usually leads to the right conclusions. Nevertheless, it is known that natural history and clinical manifestations of aortoiliac occlusive disease are influenced by its extent of distribution. Several investigators have reported impotence, buttock claudication, gluteal and/or anorectal necrosis, and paralysis of the lower extremities as a result of acute ligation or thrombosis of patent hypogastric arteries during aortoiliac bypass procedures [4]. Theoretically, the same clinical picture might present as a consequence of AIOD and mimic cauda equina syndrome [5]. Vascular examination remains the most important tool to differentiate between claudication of vascular and neurological origins. We illustrate a case of acute AIOD presenting with spinal cord ischemia and gluteal and scrotal necroses, which was initially diagnosed and treated as compressive myelopathy. Written consent was obtained from the patient prior to submission of this case report.

## 2. Case Report

**Figure 1:**
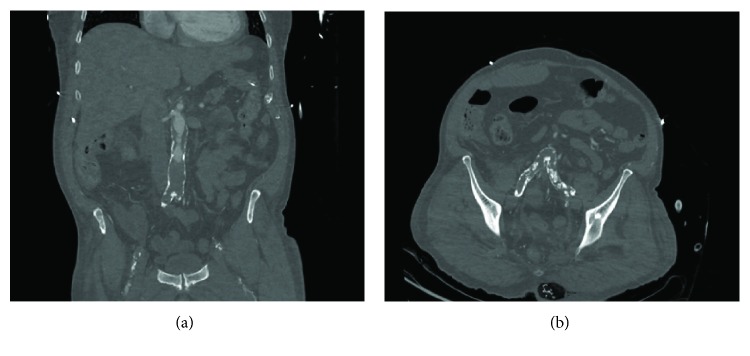
The CT angiography scan revealed extensive aortoiliac occlusion. (a) Occlusion of the distal aorta. (b) The thrombus extends into the iliac arteries.

A 72-year-old man with a long history of smoking was referred to the emergency department on suspicion of cauda equina syndrome. Mild spinal stenosis of the L4/L5 and presacral level had been found on MRI previously during a workup for moderate claudication. Other preexistent medical conditions included hypertension, diabetes, and obesity. On the previous day, his back pain had acutely worsened; difficulty in urinating and fecal incontinence had appeared. The patient had not been able to walk properly. On examination, acute urinary retention was found, as well as diminished anal sphincter function and saddle anesthesia. There was no weakness or sensory loss in either legs. The pain was localized in the lower back. The inflammation markers were markedly elevated, and the patient was feverish. Large ulcers were found in the scrotum and in the gluteal area. No vascular examination was performed. MRI of the lumbar spine was requested, and a severe spinal cord stenosis of the L4-L5 and L5-S1 levels was found. An urgent decompression procedure was performed on the same day. L5 hemilaminectomy, L4 laminotomy, and partial S1 laminotomy were performed. The findings were consistent with a moderate spinal cord stenosis. Septic shock developed during surgery, and the patient was admitted to the ICU after completion of decompression. Extensive necrotic tissue debridement was undertaken. Bacteriological cultures of samples showed polymicrobial flora, consistent with Fournier's gangrene. The patient's clinical condition did not improve, and later in ICU, both legs were found to be cold to the touch; skin appeared “marble” white. Femoral pulses were not palpable, and the skin of the lower extremity was cyanotic. A vascular surgeon was consulted, and CT angiography of the aorta and lower limbs was requested. The findings were consistent with acute on chronic aortoiliac occlusion (Figure 1).

An urgent embolectomy through bilateral groin incision was performed. Because of extensive disease of the iliac arteries, the embolectomy was not successful, and an aortobifemoral bypass with Y-prosthesis was performed. Circulation was successfully restored to the legs. Several debridement procedures and skin graft were later needed to repair the extended skin and subcutaneous necrosis in the gluteal and scrotal areas. Postoperatively, the right groin surgical wound developed superficial seroma, which was successfully treated surgically with debridement. Closure was achieved through a sartorius muscle flap. Later, the recovery was complicated by acute coronary syndrome, which required a coronary artery bypass. Eventually, the patient's conditions improved, and he was transferred to a secondary center, where his recovery continued uneventfully. After a long hospitalization, the patient underwent physical rehabilitation. The gluteal and scrotal areas needed several interventions and were eventually reconstructed with a muscular flap. The patient is currently able to walk, does not need urinary catheterization, and lives at home.

## 3. Discussion

In the case presented, the patient's long history of back pain and the previously diagnosed mild spinal cord stenosis worked as confounding factors. Vascular examination was overlooked both in the referring and in the tertiary center. When the patient presented with acute urinary retention and diminished anal sphincter function, cauda equina syndrome was immediately suspected. The MRI confirmed a worsening of the stenosis, and decompression was performed. Although signs and symptoms consistent with Fournier's gangrene were present already in the emergency department, this was not attributed to a vascular problem and was addressed only later.

Other authors have already highlighted the importance of vascular evaluation in patients presenting with paraplegia or cauda equina syndrome [6]. The absence of femoral pulses on vascular examination often aids in the diagnosis. In the case presented here, bilateral aortoiliac occlusion possibly diminished the blood flow to the pelvic region and to the lumbosacral plexus resulting in the confounding clinical picture.

Fournier's disease is a potentially fatal acute, gangrenous infection of the scrotum, penis, or perineum associated with a synergistic bacterial infection of the subcutaneous fat and superficial fascia [7]. The infection usually develops from a skin or mucosal breach. The risk factors include obesity, alcoholism, diabetes, and renal insufficiency. Ischemia plays an important role in the extension of the infection. We believe that the initial insult in our patient was of ischemic origin. The necrotic tissue became then infected, with obesity playing as a risk factor. We searched the literature for previous reports of Fournier's gangrene associated with AIOD and found only a case report from Coruh et al. [8].

Clearly, it is not possible to determine whether the clinical picture was due only to AIOD, progression of spinal compression, or a combination of both. Either way, we believe this case to be of educational value, as it is a good example of the importance of vascular examination in the emergency department. In order to avoid similar errors, careful examination of peripheral pulses of both lower limbs should always be part of the initial evaluation of cauda syndrome and paraplegia and possibly also when Fournier's gangrene is suspected.
